# Engineering Lung-on-a-chip microdevices for respiratory disease modelling and drug testing: A fit-for-purpose framework for design and translational validation

**DOI:** 10.1007/s10544-026-00849-3

**Published:** 2026-09-05

**Authors:** Sum Yi Cheong, Trevors In Zen Liew, Chee Kin Wong, Xue Xin Teng, Xin Yee Cha, Nancy Choon-Si Ng, Rebecca Shin-Yee Wong, Bey Hing Goh

**Affiliations:** 1 Department of Biomedical Sciences, Jeffrey Cheah Sunway Medical School, Faculty of Medical and Life Sciences, Sunway University, Sunway City, Malaysia; 2 Department of Medical Education, Jeffrey Cheah Sunway Medical School, Faculty of Medical and Life Sciences, Sunway University, Sunway City, Malaysia; 3 Graduate School of Medicine, KPJ Healthcare University, Nilai, Malaysia; 4 Faculty of Medicine, University of Cyberjaya, Cyberjaya, Malaysia; 5 Centre for Learning and Teaching, University of Cyberjaya, Cyberjaya, Malaysia; 6 Faculty of Health, Australian Research Centre in Complementary and Integrative Medicine, University of Technology Sydney, Ultimo, NSW Australia; 7 Sunway Biofunctional Molecules Discovery Centre, Faculty of Medical and Life Sciences, Sunway University, Sunway City, Malaysia; 8 Graduate Institute of Cancer Biology and Drug Discovery, College of Medical Science and Technology, Taipei Medical University, Taipei, Taiwan

**Keywords:** Engineering validation, Lung-on-a-chip, Microfluidics, Respiratory disease modelling, Translational validation

## Abstract

Lung-on-a-chip (LoAC) technology has emerged as a human-relevant microphysiological approach for respiratory disease modelling and preclinical drug evaluation. However, substantial variation in device architecture, membrane properties, fluidic conditions, mechanical actuation, cellular composition, sensing and manufacturing limits cross-platform comparison and translational confidence. This structured narrative review examines LoAC systems from a fit-for-purpose engineering perspective, emphasising how quantitative design parameters influence biological performance within defined contexts of use. Recent platforms demonstrate application-dependent trade-offs in membrane and interface design, flow and shear conditions, breathing-related strain, cellular complexity, analytical accessibility, scalability and reproducibility. Evidence from cancer, inhalation toxicology, infection and radiation-injury models further shows that engineering choices can alter barrier function, inflammatory responses, cellular differentiation and therapeutic sensitivity rather than merely improve physiological resemblance. To support practical assessment of translational readiness, we propose an evidence-gated framework comprising engineering verification, biological qualification, disease or pharmacological validation, human concordance, and deployment and regulatory readiness. Importantly, physiological resemblance is distinguished from demonstrated concordance with patient-derived or clinical data and, where required by the context of use, from clinically anchored predictive performance for therapeutic or toxicological outcomes. Translation will require predefined context-of-use (CoU) criteria, quantitative engineering specifications, appropriate reference comparators, clinically anchored benchmarking, quality-controlled manufacturing, standardised reporting and inter-laboratory reproducibility. Prioritising validated, fit-for-purpose performance over maximal complexity may provide a more credible pathway for advancing LoAC platforms toward reliable respiratory research, drug development and regulatory decision-support applications.

## Introduction

Respiratory drug development requires preclinical models that reproduce human pulmonary responses with sufficient physiological relevance and experimental control to support toxicity and therapeutic evaluation. Conventional two-dimensional cultures lack organised tissue interfaces, fluid dynamics and mechanical forces, whereas animal models are limited by interspecies differences that can reduce human predictivity (Petpiroon et al. 2023; Shrestha et al. 2020; Sone and Gotoh 2025).

Lung-on-a-chip (LoAC) platforms combine microfabrication and microfluidic control with pulmonary cell culture to reproduce selected features of the respiratory microenvironment, including epithelial–endothelial interfaces, air–liquid interface culture, vascular perfusion, breathing-related strain and extracellular, immune or stromal components (Huh et al. 2010; Low et al. 2021). Recent developments have expanded this design space through alternative membrane and ECM configurations, anatomically scaled structures, immune-competent and patient-derived models, controlled inhalational exposure and formats intended to improve analytical accessibility or throughput (Barata et al. 2025; Hajari et al. 2025; Licciardello et al. 2024; Rae et al. 2025; Ringquist et al. 2025).

Increasing biological or architectural complexity, however, does not necessarily improve model validity. Membrane properties, channel dimensions, materials, flow, mechanical actuation, cell source and measurement strategy can alter the generated microenvironment and consequently influence disease phenotypes and therapeutic responses (Doryab and Groll 2023; Min et al. 2025; Nawroth et al. 2023). These choices create application-dependent trade-offs between barrier compartmentalisation and immune migration, fluidic precision and operational simplicity, or miniaturisation and sample recovery. LoAC performance therefore cannot be judged by physiological resemblance or technological sophistication alone.

Although recent reviews have described LoAC technologies and applications comprehensively, practical guidance linking quantitative engineering specifications to biological performance and translational evidence remains needed (Low et al. 2021; Sone and Gotoh 2025). This review adopts a fit-for-purpose engineering perspective, examining architecture and membrane properties, fluidics, mechanical actuation, cellular and extracellular composition, sensing, manufacturing and operability. These factors are evaluated against biological and therapeutic outcomes through a staged framework encompassing engineering verification, biological qualification, disease or pharmacological validation, human concordance, and deployment and regulatory readiness. The aim is to identify the design and validation principles required to advance LoAC platforms from physiologically representative models toward reproducible preclinical and regulatory decision-support tools.

## Review scope and fit-for-purpose engineering framework

### Literature search and study selection

This structured narrative review was informed by a targeted literature search of PubMed/MEDLINE, Scopus and Web of Science Core Collection covering publications from January 2010 to August 2026, with particular emphasis on studies published from 2023 onward. The search combined terms relating to respiratory organ-on-chip systems (“lung-on-a-chip”, “airway-on-a-chip”, “alveolus-on-a-chip” and “pulmonary microphysiological system”) with engineering and translational concepts including microfluidics, architecture, membrane, material, flow, shear stress, mechanical strain, actuation, sensing, fabrication, throughput, reproducibility, standardisation, validation, qualification and context of use. Additional targeted searches addressing organ-on-chip manufacturing, quality management and regulatory translation were performed using broader “organ-on-chip”, “microphysiological system” and “new approach methodology” terminology. Reference lists of relevant reviews and primary studies were also screened to identify additional publications.

Eligible publications included human respiratory LoAC studies reporting measurable engineering specifications, studies demonstrating effects of engineering design on biological, disease, toxicological or pharmacological performance, and studies contributing evidence relevant to human concordance, reproducibility, manufacturing, scalability or translation. Recent reviews, standards-oriented publications and authoritative regulatory guidance were included where they informed cross-cutting engineering or validation principles. Foundational studies published before 2023 were retained when they established design concepts necessary for interpretation of subsequent platforms. Studies were excluded when they concerned non-respiratory systems without relevance to general validation or manufacturing principles, lacked substantive engineering or translational information, or provided insufficient methodological detail for the review objectives. Conventional cell, organoid or animal studies were not included as LoAC evidence, although selected human patient-derived models were used explicitly as comparators for clinically anchored predictive validation.

Representative platforms included in Table 1 were purposively selected from the eligible literature to illustrate the range of contemporary LoAC engineering configurations and validation evidence rather than to provide an exhaustive catalogue. Selection prioritised studies reporting quantitative device parameters and collectively representing variation in membrane/interface design, fluidic strategy, mechanical actuation, cellular complexity, analytical accessibility and scalability. Platforms were additionally selected to illustrate different levels of engineering verification, biological qualification, disease or pharmacological validation and human concordance. Recent studies were prioritised, while seminal earlier platforms were retained where necessary to establish foundational engineering comparisons.

**Table 1:** Quantitative comparison of engineering specifications, biological performance, validation level and translational limitations of representative lung-on-a-chip platforms

Context of use	Architecture and membrane/interface	Cellular configuration	Quantitative flow and mechanical conditions	Monitoring / analytical readouts	Biological endpoints / performance	Highest validation level demonstrated and principal translational limitation	References
Alveolar–capillary modelling	Two-channel alveolar–capillary interface separated by a flexible porous PDMS membrane	Alveolar epithelial cells and pulmonary microvascular endothelial cells	Vascular perfusion; vacuum-driven cyclic deformation producing ~ 10% strain at 0.2 Hz	Barrier permeability; microscopy; inflammatory and cellular responses	Breathing-related mechanical forces altered barrier function, inflammatory responses and particle-associated injury	Biological qualification. Established that controlled mechanical inputs alter barrier and cellular responses. Translation remains limited by the planar PDMS architecture, external actuation and absence of disease-specific human predictive or deployment validation.	Huh et al. (2010)
Dynamic airway maturation	Two-channel airway chip with flexible porous PDMS membrane; ~7-µm pores	Differentiated primary human airway epithelium	Medium perfusion 30 µL/h; airflow 1.2 µL/s; airflow shear ~ 0.1 mPa; cyclic stretch 5% at 0.25 Hz	Mucociliary differentiation and clearance; inflammatory and ECM-related responses	Dynamic culture accelerated epithelial maturation; airflow and stretch enhanced directional mucociliary clearance and altered inflammatory/ECM responses	Biological qualification. Quantitatively controlled airflow and strain were linked to functional epithelial outcomes. Large pores permitted unintended epithelial migration and required surface optimisation; human concordance and predictive validation were not demonstrated.	Nawroth et al. (2023)
Radiation-induced lung injury and therapeutic testing	Human alveolus chip reproducing an epithelial–vascular interface with flexible porous membrane	Human alveolar epithelial cells and lung microvascular endothelial cells	Vascular perfusion with breathing-related cyclic strain	DNA damage; barrier integrity; inflammatory signalling; therapeutic responses	Reproduced dose-dependent radiation injury and enabled evaluation of prednisolone and lovastatin; radiation sensitivity showed greater relevance to reported human responses than conventional models	Disease/pharmacological validation. Demonstrated disease-relevant injury and treatment responses within a defined CoU. Direct patient-level predictive validation, independent replication and broader transferability remain unestablished.	Dasgupta et al. (2023)
Miniaturised multicellular lung interface	Miniaturised multilayer platform with electrospun PCL–gelatin membrane and collagen-based interstitial matrix	Epithelial and endothelial compartments with fibroblasts embedded within ECM	Microfluidic perfusion; breathing actuation not the principal design feature	Microscopy; multicellular and tissue-interface analyses	Reproduced epithelial, interstitial and vascular organisation while incorporating a fibroblast-containing ECM compartment	Biological qualification. Demonstrated a multicellular tissue interface with reduced cell and reagent requirements. Multilayer fabrication increases manufacturing complexity, while disease-specific, human-concordance and inter-laboratory evidence remain limited.	Licciardello et al. (2024)
Immunocompetent alveolar modelling	Compartmentalised alveolar epithelial–endothelial interface under ALI conditions	Alveolar epithelial cells, endothelial cells and primary monocyte-derived macrophages	Continuous perfusion 21 µL/min; mechanical actuation not the principal variable	Barrier; inflammatory and immune responses	Macrophage incorporation enabled evaluation of innate immune responses within a perfused alveolar microenvironment	Biological qualification. Functionally integrated resident immune components. Primary immune-cell sourcing introduces donor and culture variability, and direct human benchmarking, predictive performance and inter-laboratory reproducibility remain limited.	Koceva et al. (2024)
High-containment respiratory infection modelling	Sealed airway platform; 10-µm PET membrane, 0.4-µm pores, ~ 6 × 10^6^ pores/cm^2^	Primary human bronchial epithelial cells in compartmentalised culture	Pump-free gravity-driven bidirectional perfusion generated by rocking; no dedicated breathing actuator	Epithelial differentiation; barrier-related and infection-associated molecular readouts	Maintained differentiated airway culture and supported controlled respiratory viral studies without external pumping infrastructure	Biological qualification. Simplified pump- and tubing-free operation supports containment and usability. Less precise instantaneous flow/shear control and fabrication-dependent ALI stability remain important limitations; broader predictive and inter-laboratory validation are lacking.	Barata et al. (2025)
Large-area airway modelling and analytical recovery	Large-area airway platform; culture area ~ 92 mm^2^; 23-µm PET membrane with 0.4-µm pores	Differentiated human airway epithelium with supporting compartment	Perfusion 150 µL/h; calculated shear stress ~ 5 × 10^− 4^ dyn/cm^2^; cyclic strain not required	RNA, protein and secreted-factor recovery; microscopy; epithelial differentiation	Supported long-term epithelial differentiation while generating sufficient biological material for conventional downstream assays	Biological qualification. Improved analytical accessibility and compatibility with conventional workflows. The intentionally low shear does not reproduce physiological vascular mechanics, and predictive or independent inter-laboratory validation remains to be demonstrated.	Rae et al. (2025)
Immune-competent severe influenza modelling	Multicellular airway–interstitial–vascular platform with perfusable microvasculature; ~5-µm membrane pores permitting immune migration	Airway epithelium, fibroblasts, vascular cells, tissue-resident macrophages and circulating immune cells	Perfusable vascular compartment; breathing-related mechanical actuation not the principal variable	Cytokine profiles; immune-cell recruitment; comparison with patient BAL responses	Resident macrophages improved concordance with patient inflammatory responses; broader immune integration reproduced features of severe influenza-associated cytokine responses	Human concordance. On-chip inflammatory responses were benchmarked against patient-derived BAL data. High cellular complexity increases sourcing and operational burden, while clinical predictive performance and inter-laboratory reproducibility remain unestablished.	Ringquist et al. (2025)
Whole-cigarette-smoke inhalation toxicology	Breathing LoAC integrated with whole-smoke delivery under ALI conditions and a deformable interface	Airway epithelial model	Cyclic strain 10% at 0.2 Hz with controlled whole-cigarette-smoke exposure	Serial TEER; cytotoxicity; barrier disruption; IL-8 expression	Whole smoke reduced barrier resistance by ~ 60% and increased IL-8 expression ~ 4.5-fold; dynamic strain accelerated barrier disruption relative to static conditions	Disease/toxicological validation. Demonstrated exposure-responsive barrier and inflammatory effects under controlled respiratory mechanics. Exposure generation and actuation increase operational complexity, and direct human-outcome prediction has not been established.	Sengupta et al. (2025)
Anatomically scaled alveolar mechanics	Interconnected ~ 500-µm alveolar chambers with ~ 150-µm interconnections on a deformable membrane; compatible with 12- or 24-well formats	Alveolar cellular configuration	Pneumatic actuation producing ~ 5–15% linear strain with pressure–deformation calibration	Deformation mapping; mechanical stability; biological assessment	Enabled controlled three-dimensional deformation of anatomically scaled alveolar structures during cyclic operation	Engineering verification. Quantified and calibrated mechanical deformation while improving multiwell parallelisation. The nonporous deformable membrane does not reproduce transport across a porous air–blood interface, and disease-specific, human-concordance and deployment evidence remain limited.	Hajari et al. (2025)

Notes: Validation level indicates the highest level of evidence demonstrated in the cited study according to the fit-for-purpose framework proposed in this review and does not imply formal regulatory qualification or acceptance. Quantitative values are reported as described in the cited studies and should not be interpreted as universal acceptance thresholds; appropriate specifications and acceptance criteria depend on the intended context of use. Abbreviations: *ALI* air–liquid interface, *BAL* bronchoalveolar lavage, *ECM* extracellular matrix, *IL-8* interleukin-8, *LoAC* lung-on-a-chip, *PCL* polycaprolactone, *PDMS* polydimethylsiloxane, *PET* polyethylene terephthalate, *TEER* transepithelial electrical resistance

### Fit-for-purpose analytical framework

**Fig. 1:**
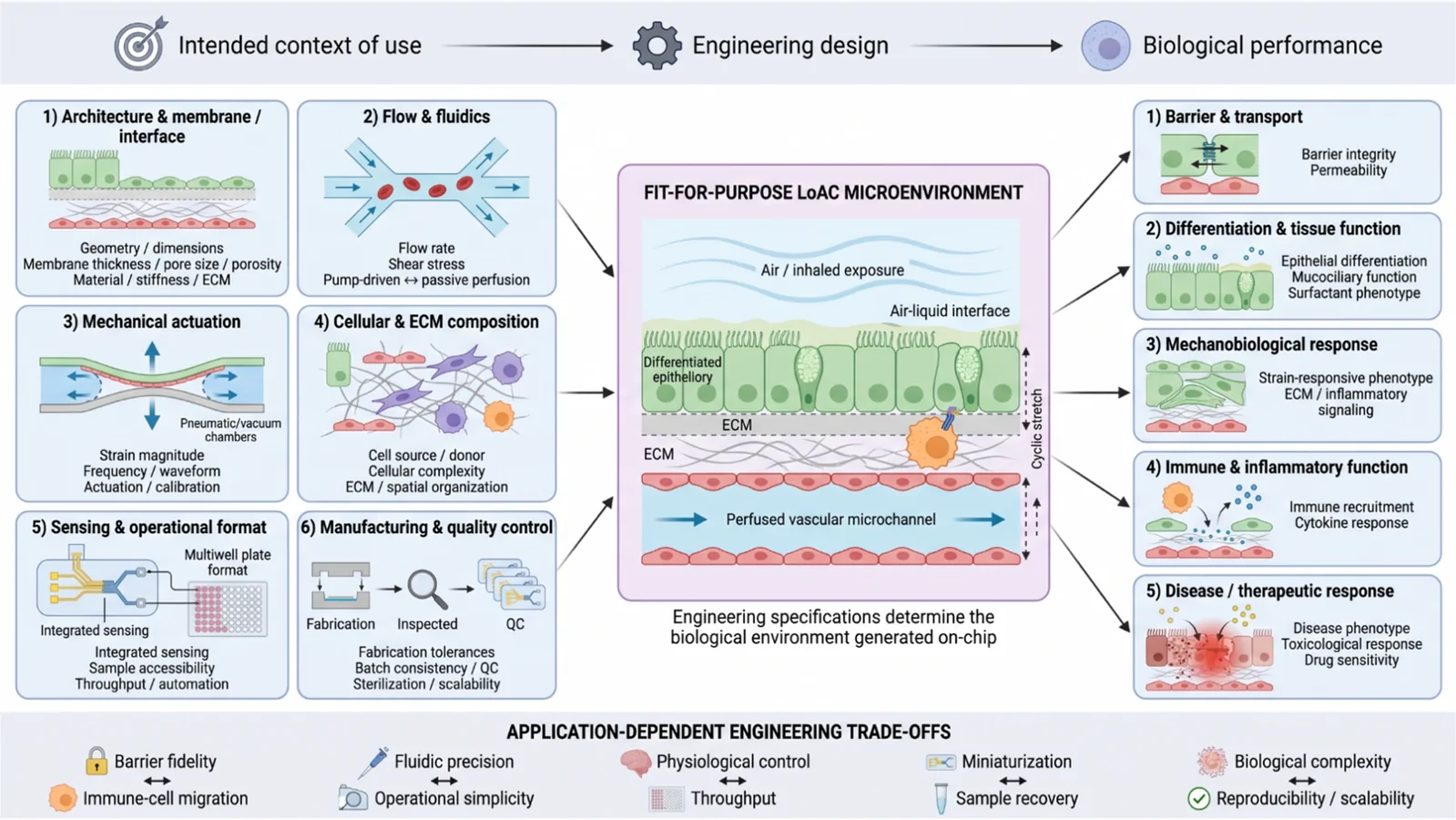
Intended context of use–guided engineering design of lung-on-a-chip (LoAC) microdevices and its relationship to biological performance. The schematic illustrates how the intended context of use informs key engineering design domains—architecture and membrane/interface properties, flow and fluidics, mechanical actuation, cellular and extracellular matrix (ECM) composition, sensing and operational format, and manufacturing and quality control—to generate a fit-for-purpose LoAC microenvironment. This engineered microenvironment, in turn, shapes major biological outputs, including barrier and transport function, differentiation and tissue function, mechanobiological responses, immune and inflammatory function, and disease or therapeutic responses. The figure also highlights application-dependent engineering trade-offs, including barrier fidelity versus immune-cell migration, fluidic precision versus operational simplicity, physiological control versus throughput, miniaturization versus sample recovery, and biological complexity versus reproducibility/scalability

The literature was analysed according to a fit-for-purpose framework in which engineering specifications were evaluated in relation to the biological function and intended context of use. Engineering variables included architecture and dimensions, membrane and material properties, flow and shear conditions, mechanical strain, cellular and extracellular composition, sensing strategy, manufacturing and operational format. Their consequences were assessed through corresponding functional outcomes, including barrier performance, epithelial differentiation, mucociliary activity, immune recruitment, inflammatory responses and therapeutic or toxicological effects. The relationship between intended context of use, engineering design specifications and the resulting biological performance of LoAC systems is summarised in Fig. 1.

Translational evidence was subsequently evaluated across five stages: engineering verification, biological qualification, disease or pharmacological validation, human concordance, and deployment and regulatory readiness. The framework therefore distinguishes technological or physiological resemblance from demonstrated performance and evaluates whether each platform provides sufficiently defined, reproducible and biologically appropriate evidence for its intended context of use.

## Engineering parameters governing lung-on-a-chip performance

LoAC performance is determined by interacting architectural, material, fluidic, mechanical and biological variables rather than any single design feature. Contemporary platforms vary substantially in membrane configuration, channel geometry, perfusion, mechanical actuation, cellular complexity and analytical format because these elements are selected for different contexts of use rather than converging toward one optimal design. Engineering specifications should therefore be judged according to the function that the platform is intended to reproduce. Representative LoAC platforms are compared in Table 1 according to quantitative engineering specifications, biological endpoints, the highest validation level demonstrated and principal translational limitations.

### Architecture, membranes and biological interfaces

Conventional LoAC systems commonly comprise epithelial and endothelial compartments separated by a porous membrane that supports spatial organisation of the air–blood interface while permitting molecular exchange and, depending on pore size, cellular communication (Huang et al. 2021; Huh et al. 2010). Membrane thickness, pore size, porosity, stiffness and ECM functionalisation are not passive structural features; they influence transport, attachment, mechanical deformation and intercompartmental signalling (Doryab and Groll 2023; Min et al. 2025).

Recent platforms illustrate the resulting trade-offs. Small-pore PET membranes can favour compartmentalised epithelial culture, whereas larger pores may be selected to permit immune-cell transmigration, demonstrating that barrier fidelity and leukocyte recruitment may require different interface properties (Barata et al. 2025; Rae et al. 2025; Ringquist et al. 2025). Alternative designs use electrospun polycaprolactone–gelatin membranes and fibroblast-containing collagen matrices to introduce a more tissue-like interstitial compartment (Licciardello et al. 2024).

Architecture is similarly application-dependent. Planar channels facilitate fabrication, imaging and barrier measurements but simplify distal-lung geometry. Anatomically scaled alveolar structures can improve geometric and mechanical representation, although deformable nonporous interfaces may sacrifice the trans-barrier transport achieved with porous membranes (Hajari et al. 2025). Membrane and compartment design should therefore be selected according to whether the intended endpoint prioritises transport, immune migration, mechanical deformation, analytical accessibility or combinations of these functions (Barata et al. 2025; Hajari et al. 2025; Rae et al. 2025).

### Flow, shear and fluidic strategy

Perfusion supports nutrient delivery, waste removal, compound transport and endothelial mechanostimulation, but volumetric flow rate alone does not define the cellular environment. Wall shear stress also depends on channel geometry, dimensions and fluid viscosity, meaning that identical flow rates can produce different mechanical exposures.

Contemporary platforms span pump-driven and passive strategies. Continuous perfusion has been used in immunocompetent alveolar systems, whereas larger airway platforms may deliberately operate at very low shear to prioritise long-term culture and sample recovery (Koceva et al. 2024; Rae et al. 2025). Pump-free gravity-driven rocking can simplify operation and reduce tubing-related contamination risks in high-containment infection studies, although it provides less precise control over instantaneous flow and shear (Barata et al. 2025).

Fluidic complexity should therefore be matched to the experimental question. Pump-driven systems offer greater control over flow profiles and compound delivery but increase dead volume, bubble risk and operational burden, while passive systems improve accessibility at the expense of mechanical precision. Studies should report flow-generation method, channel dimensions and, where relevant, calculated or measured shear stress to distinguish perfusion-mediated transport from endothelial mechanostimulation (Koceva et al. 2024; Rae et al. 2025).

### Mechanical actuation and respiratory biomechanics

Mechanical stimulation is a defining capability of LoAC technology, but “breathing motion” encompasses different strain magnitudes, frequencies, waveforms and actuation mechanisms. The original alveolus chip established vacuum-driven membrane deformation as a means of reproducing breathing-related strain (Huh et al. 2010), while subsequent work has treated mechanical stimulation as a quantitatively defined experimental variable rather than a simple dynamic-versus-static condition (Kaarj and Yoon 2019; Shiraishi et al. 2023).

Recent studies demonstrate that these parameters influence biological outcomes. Cyclic stretch combined with airflow and perfusion can accelerate mucociliary maturation and alter inflammatory and ECM-related responses, whereas dynamic strain can modify cellular responses to inhaled toxicants (Nawroth et al. 2023; Sengupta et al. 2025). Pressure–deformation mapping has also been used to calibrate strain and assess stability during repeated actuation (Hajari et al. 2025). These findings show that dynamic culture cannot be interpreted without specifying the magnitude and temporal characteristics of the imposed mechanical forces.

Actuation is also coupled to membrane design. Flexible membranes enable deformation, but composition, thickness and pore architecture determine strain transmission and may introduce unintended biological effects; for example, larger membrane pores can permit epithelial migration during dynamic culture (Nawroth et al. 2023). Mechanical stimulation should therefore be reported using strain magnitude, frequency, waveform, actuation method and calibration, with spatial characterisation where deformation is heterogeneous (Huh et al. 2010; Kaarj and Yoon 2019; Nawroth et al. 2023; Sengupta et al. 2025).

### Cellular and extracellular microenvironment

Cellular complexity should be treated as a design variable rather than an implicit indicator of model quality. Epithelial–endothelial co-cultures provide a tractable respiratory-barrier model, whereas incorporation of fibroblasts, macrophages, circulating leukocytes or patient-derived cells enables investigation of tissue remodelling, immune recruitment and inter-individual responses (Lagowala et al. 2021; Plebani et al. 2022; Sengupta et al. 2022). Additional cell populations, however, introduce requirements for compatible media, ECM composition, spatial organisation and culture duration that can increase variability.

Immune-competent platforms illustrate why complexity should be justified functionally. Models incorporating monocyte-derived macrophages, fibroblasts, microvasculature and circulating immune cells have demonstrated functionally richer inflammatory and immune-recruitment responses, including improved concordance of selected cytokine profiles with patient bronchoalveolar lavage data (Koceva et al. 2024; Ringquist et al. 2025). The design objective should therefore be the minimum biological complexity required for the intended function, rather than the greatest possible number of cell types.

Primary and patient-derived cells may increase physiological specificity but introduce donor variability and limited expansion, whereas immortalised cells improve standardisation at the cost of some phenotypic fidelity (Lagowala et al. 2021; Sengupta et al. 2022). Cell source, donor characteristics, passage, seeding strategy and phenotype verification should consequently be treated as part of the platform specification.

### Sensing, operability and throughput

LoAC utility also depends on whether biological outputs can be measured reproducibly and whether the system can be operated using practical laboratory workflows. Electrical barrier measurements, permeability assays, microscopy and molecular analyses provide complementary information, but their feasibility depends on electrode configuration, sampling volume, culture area and analytical compatibility (Benam et al. 2016; Y. Wang et al. 2023a).

Different platforms address these requirements in contrasting ways. Serial electrical-resistance measurements permit longitudinal monitoring during inhalational exposure, while larger culture areas improve RNA, protein and secreted-factor recovery for conventional assays (Rae et al. 2025; Sengupta et al. 2025). Miniaturised platforms reduce cell and reagent use and may improve parallelisation, whereas sealed pump-free devices and multiwell-compatible alveolar systems simplify containment or experimental scaling (Barata et al. 2025; Hajari et al. 2025; Licciardello et al. 2024).

These strategies reveal a trade-off between miniaturisation, analytical accessibility, physiological control and throughput. Smaller systems reduce resource use but may limit sample recovery, while highly controlled or biologically complex devices can increase operational burden and hinder automation. The most useful design is therefore the platform whose monitoring strategy, operation and analytical outputs are matched to its intended application rather than the most technologically elaborate system.

### Manufacturing, quality control and reproducibility

Reproducible biological performance requires reproducible manufacture. Variation in channel dimensions, membrane position, bonding, surface properties, actuator geometry or fluidic resistance can alter shear stress, strain transfer, exposure delivery and barrier behaviour. Critical device attributes should therefore be accompanied by predefined tolerances and functional quality-control criteria, including dimensional conformity, leak tightness, flow accuracy, surface treatment, sensor calibration and actuator performance (Pamies et al. 2024; Piergiovanni et al. 2021).

Quality management must also encompass membranes, ECM preparations, coatings, cells, reagents and operating procedures. Batch traceability, acceptance criteria and documentation of fabrication parameters, equipment and operators are important because technical and biological variability can otherwise become indistinguishable. Reproducibility should be assessed across device batches, experiments, operators and, where appropriate, biological donors, supported by standard operating procedures and predefined responses to non-conformities (Pamies et al. 2024).

Manufacturing strategy introduces further trade-offs. Soft lithography and PDMS remain useful for flexible prototyping but are less suited to high-volume production. Thermoplastic replication methods such as hot embossing and microinjection moulding provide greater production reproducibility and scalability, although tooling requirements and material constraints differ (Olaizola-Rodrigo et al. 2025). Importantly, changing fabrication method or material may alter gas permeability, optical performance, surface chemistry, compound adsorption or mechanical behaviour and should therefore trigger re-verification of critical engineering and biological endpoints.

Sterilisation, automation and interoperability should likewise be incorporated into platform development. Sterilisation must preserve bonding, membrane properties, surface functionalisation, sensors and material integrity; established thermoplastic approaches include gamma irradiation and ethylene oxide treatment, although suitability remains device-specific (Olaizola-Rodrigo et al. 2025; Pamies et al. 2024). Automation can reduce operator-dependent variability but requires corresponding calibration and quality-control procedures, while standardised interfaces with pumps, imaging systems and analytical equipment can facilitate transfer between laboratories (Pamies et al. 2024; Piergiovanni et al. 2021). Manufacturing readiness should therefore be judged by the ability to reproducibly meet predefined engineering specifications and retain qualified biological performance across batches, operators and sites.

## Linking engineering design to biological and therapeutic performance

Engineering specifications become meaningful only when they produce measurable differences in biological or therapeutic performance. Across recent and foundational LoAC studies, architecture, mechanical stimulation, exposure delivery and cellular composition have altered experimental outcomes rather than simply improving visual resemblance to native lung tissue. Disease and drug-testing applications can therefore serve as functional tests of engineering validity, provided that the relationship between the imposed engineering condition and resulting biological response is explicitly demonstrated.

Mechanical context provides a clear example. In an orthotopic non-small-cell lung cancer chip, breathing-related mechanical deformation altered tumour proliferation, invasion, dormancy and responsiveness to tyrosine kinase inhibition. The system reproduced growth and treatment behaviours associated with human disease while demonstrating that physical cues related to breathing can modify epidermal growth factor receptor and MET signalling (Hassell et al. 2017). The implication extends beyond cancer modelling: therapeutic response cannot always be attributed to cellular genotype or drug exposure alone when the mechanical microenvironment itself modifies phenotype. Similarly, cyclic strain in alveolar models has altered epithelial barrier and differentiation-related responses, supporting the treatment of strain magnitude and frequency as experimentally relevant biological inputs rather than optional device features (Huh et al. 2010; Sengupta et al. 2022).

Exposure delivery can likewise determine the magnitude and type of injury detected. A breathing LoAC integrating whole-cigarette-smoke exposure, air–liquid interface culture and cyclic strain demonstrated an approximately 60% reduction in barrier resistance and a 4.5-fold increase in IL-8 expression, while dynamic strain accelerated barrier disruption and cytotoxicity relative to static conditions (Sengupta et al. 2025). Such findings illustrate that exposure engineering affects not only physiological representation but also assay sensitivity, because submerged extracts cannot reproduce the gaseous and particulate composition, deposition process and mechanical context of inhalational exposure.

Cellular configuration provides a third demonstration of engineering-dependent performance. In an immune-competent airway model, addition of tissue-resident macrophages generated cytokine-response profiles that showed greater concordance with those measured in patient bronchoalveolar lavage, whereas incorporation of resident and circulating immune populations was required to reproduce broader severe influenza-associated inflammatory responses (Ringquist et al. 2025). This functional gain depended on deliberate engineering choices, including a 5-µm-pore interface and perfusable microvasculature that permitted immune-cell migration. Biological complexity is therefore most informative when it enables a predefined function, such as leukocyte recruitment or inflammatory signalling, rather than when additional cell populations are incorporated solely to increase model complexity.

Therapeutic applications provide a further level of functional evaluation. A human alveolus chip exposed to ionising radiation reproduced epithelial and endothelial DNA damage, inflammatory signalling and barrier disruption and was subsequently used to evaluate prednisolone and lovastatin (Dasgupta et al. 2023). Together with mechanically sensitive cancer-chip studies, these findings demonstrate that the engineered microenvironment can determine the phenotype against which therapeutic activity is assessed (Dasgupta et al. 2023; Hassell et al. 2017).

Collectively, these examples support evaluating LoAC platforms according to fit-for-purpose functional performance rather than anatomical or technological complexity alone. Mechanical strain should be linked to mechanosensitive endpoints, immune integration to demonstrable recruitment or inflammatory function, and inhalational exposure to controlled delivery and exposure-responsive readouts. However, demonstration of such functional effects does not by itself establish human concordance or clinical predictivity; these stronger translational claims require progressively more stringent evidence within the proposed validation framework, including direct comparison with human reference data and, where appropriate, clinically anchored predictive performance.

## Translational validation and readiness

**Fig. 2:**
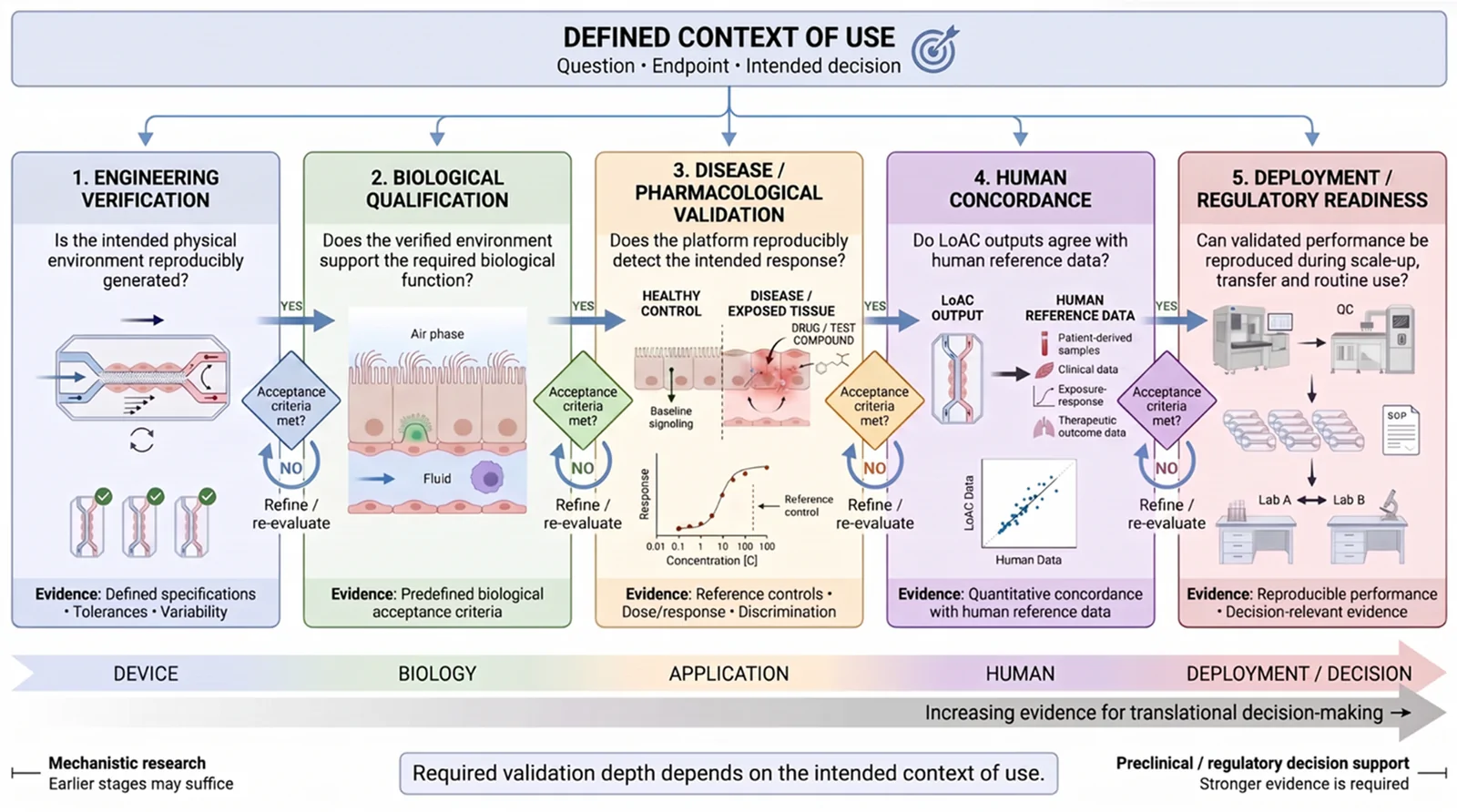
Context-of-use-driven framework for progressive validation of lung-on-a-chip (LoAC) platforms. The required depth of validation is determined by the defined context of use, including the scientific or decision question, endpoint, and intended application. The framework progresses through five stages: engineering verification, biological qualification, disease/pharmacological validation, human concordance, and deployment/regulatory readiness. At each stage, predefined acceptance criteria determine progression, while refine/re-evaluate loops emphasize the iterative nature of platform development and validation. The framework further highlights that earlier validation stages may be sufficient for mechanistic research, whereas preclinical or regulatory decision support requires stronger evidence across later stages

Demonstrating functional performance on-chip does not by itself establish translational validity. For LoAC platforms to support preclinical or regulatory decision-making, validation should be anchored to a clearly defined context of use (CoU) specifying the question addressed, intended decision, operating conditions, relevant endpoints and appropriate reference comparators. Performance requirements for mechanistic modelling, toxicity assessment and therapeutic prediction are therefore not interchangeable (Alver et al. 2024; Araújo-Gomes et al. 2026; Weener et al. 2024). Recent FDA draft guidance similarly frames NAM validation around CoU, human biological relevance, technical characterization and fitness for purpose (Food and Drug Administration, 2026). The proposed framework therefore considers five evidence-linked stages: engineering verification, biological qualification, disease or pharmacological validation, human concordance, and deployment and regulatory readiness. Progression should depend on predefined, CoU-specific acceptance criteria rather than universal thresholds. The operational criteria, representative measurable endpoints and evidence requirements for each validation stage are summarised in Table 2, while the overall context-of-use-driven progression from engineering verification through deployment and regulatory readiness is illustrated in Fig. 2.

**Table 2:** Fit-for-purpose validation criteria for lung-on-a-chip platforms

Validation stage	Core question	Example measurable criteria	Evidence required for progression
Engineering verification	Does the platform reproducibly generate the intended physical and operational environment?	Device and membrane dimensions; pore characteristics; flow/shear conditions; strain magnitude/frequency; exposure delivery; sensor calibration; leakage; operational stability	Critical engineering inputs remain within predefined CoU-specific tolerances across independent devices or fabrication batches, with variability characterised
Biological qualification	Does the verified engineering environment support the biological function required for the intended application?	Cell viability/identity; barrier integrity or permeability; differentiation; mucociliary function; surfactant-associated phenotype; endothelial or immune function	Predefined biological acceptance criteria are achieved reproducibly during the intended experimental window and, where relevant, across donors or cell batches
Disease or pharmacological validation	Does the qualified platform reproducibly detect the intended disease, exposure or therapeutic response?	Disease-relevant phenotype or biomarker; positive/negative controls; dose- or concentration-response; reference exposures/compounds; response discrimination	Target response is reproducible and distinguishable from appropriate comparator conditions using prespecified criteria; sensitivity/specificity or related metrics may be used where required by the CoU
Human concordance	Do LoAC outputs agree with appropriate patient-derived or clinical reference data?	Patient tissue/biofluid measurements; clinical biomarkers; human exposure-response relationships; therapeutic outcomes; correlation, agreement or rank-order metrics	Quantitative concordance with an appropriate human reference dataset is demonstrated using predefined endpoints or performance metrics
Deployment and regulatory readiness	Can qualified performance be maintained during scale-up, transfer and routine use, and is the evidence interpretable for the intended decision?	Manufacturing specifications; batch-release QC; material/cell traceability; validated sterilisation/storage; SOPs; automation; interoperability; inter-operator and inter-laboratory reproducibility	Previously qualified engineering and biological performance is retained across batches, operators and sites, with documented QC, reproducibility and evidence appropriate to the intended regulatory or preclinical CoU

Note: The criteria shown are illustrative and should not be interpreted as universal numerical thresholds. Appropriate acceptance criteria and the level of evidence required depend on the defined context of use. Progression is evidence-gated, and completion of an earlier stage does not establish performance at a later stage. Abbreviations: *CoU* context of use, *LoAC* lung-on-a-chip, *QC* quality control, *SOP* standard operating procedure

The first stage, engineering verification, establishes whether the platform reproducibly generates its intended physical and operational environment. Relevant measurements include channel and membrane dimensions, pore characteristics, flow and shear conditions, strain magnitude and frequency, exposure delivery, sensor calibration, leakage and stability over the experimental period. These should be evaluated across independent devices or fabrication batches against predefined tolerances rather than nominal settings alone. For example, mechanosensitive models should verify the applied strain field, whereas inhalation platforms should verify delivered exposure rather than relying solely on source concentration. Quality controls may therefore include dimensional inspection, flow verification, leak and bubble testing, sensor calibration and confirmation of actuator performance (Araújo-Gomes et al. 2026; Pamies et al. 2024). Engineering verification is achieved when critical physical inputs remain within acceptable limits and their variability is characterised.

The second stage, biological qualification, asks whether the verified engineering environment supports the biological functions required for the intended application. Appropriate endpoints may include viability, epithelial or endothelial barrier integrity, differentiation, mucociliary function, surfactant-associated phenotype or immune-cell migration. These endpoints should be selected according to the CoU rather than from a universal panel. Barrier and transport performance may be critical for permeability studies, whereas mucociliary function may be more relevant to airway exposure models and immune recruitment to infection studies. Qualification should also demonstrate stability during the intended experimental window and reproducibility across independent experiments and, where relevant, donors or cell batches (Araújo-Gomes et al. 2026; Pamies et al. 2024; Weener et al. 2024). Importantly, biological qualification establishes fitness for a specified biological function; it does not demonstrate clinical prediction.

The third stage, disease or pharmacological validation, requires the qualified platform to reproducibly detect the response for which it is intended. Disease models should reproduce prespecified disease-relevant phenotypes or biomarkers relative to appropriate comparators, while toxicological or therapeutic applications should incorporate relevant reference exposures or compounds, positive and negative controls, and dose- or concentration-response relationships where applicable. Where classification or prediction is required, metrics such as sensitivity, specificity or response discrimination may be appropriate (Araújo-Gomes et al. 2026). Existing LoAC examples include radiation-induced DNA damage, inflammation and barrier disruption followed by evaluation of candidate countermeasures (Dasgupta et al. 2023), and enhanced smoke-induced barrier injury under dynamic compared with static conditions (Sengupta et al. 2025). Progression therefore requires a reproducible target response relative to an accepted comparator, rather than biological plausibility alone.

The fourth stage, human concordance, should be distinguished explicitly from physiological resemblance and predictive performance. Reproduction of human-like architecture, air–liquid interface conditions or breathing-related mechanics may increase biological relevance, but human concordance requires direct comparison of LoAC outputs with appropriate patient-derived or clinical reference data. Depending on the CoU, this may involve patient tissue or biofluid measurements, clinical biomarkers, exposure-response relationships, therapeutic outcomes, or quantitative agreement and rank-order analyses. The immune-competent influenza LoAC provides one example in which cytokine-response profiles were compared with patient bronchoalveolar lavage data (Ringquist et al. 2025). Such evidence supports concordance for the measured inflammatory endpoints but should not automatically be interpreted as broader clinical prediction.

Predictive performance requires a stronger evidentiary standard when the CoU involves therapeutic or toxicological decision-making. Model outputs should discriminate or forecast clinically relevant outcomes using predefined metrics and appropriately characterised reference datasets; relevant measures may include sensitivity, specificity, accuracy, calibration or associations with treatment response, toxicity or survival. Patient-derived respiratory models outside the LoAC field illustrate this evidentiary level: short-term lung cancer cell drug sensitivity has been associated with targeted-therapy response and progression-free survival, while lung cancer organoid testing has demonstrated clinically anchored classification performance (Kim et al. 2024; H.-M. Wang et al. 2023b). These studies do not validate LoAC technology itself, but demonstrate the type of direct linkage between model-derived measurements and human outcomes required to support predictive claims.

The fifth stage, deployment and regulatory readiness, asks whether qualified performance can be maintained across manufacturing batches, operators, scale-up and independent laboratories, and whether the resulting evidence is sufficiently standardized and interpretable for the intended decision. Relevant requirements include defined production specifications, batch-release criteria, traceability of critical materials and biological components, validated sterilisation or storage conditions, calibrated ancillary equipment, documented standard operating procedures and inter-laboratory reproducibility (Pamies et al. 2024; Piergiovanni et al. 2021). Scalable manufacturing should preserve previously qualified engineering and biological performance; changes in material or fabrication method may therefore require bridging studies rather than being treated as neutral process changes (Olaizola-Rodrigo et al. 2025). Automation can reduce operator-dependent variability, but automated workflows similarly require adapted calibration and quality-control procedures.

A worked example illustrates how these stages may be applied to an inhalation-toxicology CoU. For a breathing LoAC intended to evaluate cigarette-smoke-induced airway injury, engineering verification would require reproducible smoke delivery and confirmation of the specified cyclic strain and operating conditions. Biological qualification would establish stable epithelial barrier and differentiated airway function before exposure. Disease or toxicological validation would then require reproducible smoke-responsive endpoints relative to appropriate controls; for example, Sengupta et al. (2025) reported approximately 60% reduction in barrier resistance and a 4.5-fold increase in IL-8 expression under whole-smoke exposure. These findings support toxicological validation for the measured endpoints but do not establish human concordance. Progression to that stage would require comparison with corresponding human exposure or clinical data, while deployment or regulatory readiness would additionally require preservation of qualified performance across fabrication batches, operators and independent laboratories. Thus, the framework identifies both the evidence demonstrated and the additional evidence required for the intended CoU.

Regulatory translation also requires distinction between validation, qualification and regulatory acceptance. Validation establishes that a method produces reliable and relevant outputs for its intended purpose, whereas qualification provides a formal conclusion that its results can be interpreted and used within a specified CoU. Definitions vary between regulatory and standard-setting organisations, but they converge on the principles of reliability, relevance and fitness for purpose (Araújo-Gomes et al. 2026). FDA draft guidance further indicates that both qualified and non-qualified NAMs may contribute to regulatory decision-making when their use is appropriately justified within the specific context and overall weight of evidence (Food and Drug Administration, 2026). Consequently, regulatory credibility should not be equated with universal formal qualification.

Standardisation should likewise focus on performance and reporting rather than identical device architecture. Different LoAC applications legitimately require different geometries, materials and operating conditions. More useful targets for harmonisation include terminology, reporting of critical engineering and biological parameters, predefined quality-control procedures, reference controls, analytical methods, interoperability and assessment of repeatability and inter-laboratory reproducibility (Pamies et al. 2024; Piergiovanni et al. 2021). This approach enables comparison and transferability without constraining platform-specific innovation.

Progression through this framework should therefore be evidence-gated rather than complexity-driven. Engineering reproducibility does not establish biological fitness; biological qualification does not establish human concordance; concordance does not necessarily demonstrate therapeutic or toxicological prediction; and predictive performance within one dataset does not establish deployment or regulatory readiness. The appropriate validation endpoint should instead be determined by the defined CoU (Alver et al. 2024; Araújo-Gomes et al. 2026; Weener et al. 2024). Mechanistic research models may require only engineering verification and biological qualification, whereas platforms intended to inform preclinical or regulatory decisions require progressively stronger evidence. As practical next steps, developers should define the CoU and acceptance criteria before experimentation, report critical engineering parameters and their tolerances, benchmark biological responses against prespecified reference comparators, incorporate human datasets when predictive claims are intended, and test whether qualified performance is retained across batches, operators and laboratories (Low et al. 2021; Pamies et al. 2024; Piergiovanni et al. 2021). These actions provide a practical route for advancing LoAC platforms from specialised experimental systems toward credible preclinical and regulatory decision-support tools.

## Conclusion

LoAC translation depends less on maximal biological or technological complexity than on whether engineering specifications reproducibly generate the functions required for a defined context of use. Architecture, membrane properties, fluidics, mechanical actuation, cellular composition, sensing and manufacturing therefore need to be evaluated as interdependent determinants of biological and therapeutic performance.

Progress toward preclinical or regulatory decision support should be evidence-gated, advancing from engineering verification and biological qualification to disease or pharmacological validation, human concordance and, where required by the context of use, clinically anchored predictive performance and deployment readiness. Standardised reporting, quality-controlled manufacturing, appropriate reference comparators and inter-laboratory reproducibility will be central to this progression. A fit-for-purpose approach that links engineering design to predefined performance criteria provides a more credible basis for translating LoAC platforms into reliable respiratory research and drug-development tools.

## Data Availability

No datasets were generated or analysed during the current study.
